# Synthesis, characterization, and anticancer activity of tuna bone gelatin peptide-stabilized selenium nanowires

**DOI:** 10.1039/d5ra06829k

**Published:** 2025-12-09

**Authors:** Muhammad Babar Khawar, Kamaran Khursheed Dar, Ali Afzal, Aisha Munawar, Muhammad Waqas Ishaq, Fakhar Zaman

**Affiliations:** a Applied Molecular Biology and Biomedicine Lab, Department of Zoology, University of Narowal Narowal Pakistan; b Department of Zoology, University of Azad Jammu and Kashmir Pakistan; c Molecular Medicine and Cancer Therapeutics Lab, Department of Zoology, Faculty of Science and Technology, University of Central Punjab Lahore Pakistan; d Department of Chemistry, University of Engineering and Technology Lahore 39161 Pakistan fakhar86@gmail.com mwaqasmayo@utexas.edu

## Abstract

Organic selenium shows promise in cancer treatment due to its antioxidant properties, however, challenges like toxicity and instability hinder its efficacy. Herein, we aim to develop stable and less toxic selenium-chelated tuna bone gelatin peptides (TBGP@Se). TBGPs were extracted from tuna bones and chelated with selenium through a redox reaction. The resulting TBGP@Se was characterized to assess amino acids sequences, morphology of TBGP@Se, particle size distribution. Antiproliferation activity was evaluated using A549 and HT-29 cell lines. Our TBGP@Se was rich in glycine, proline, and alanine which aided stable Se chelation. Electron micrography confirmed gelatin concentrations (5 mg mL^−1^) with stable TBGP@Se complexes. FTIR and DLS analyses further confirmed successful Se chelation and improved particle dispersion. TBGP@Se exhibited potent antiproliferative effects *in vitro*. Collectively, our study demonstrates the successful synthesis of stable TBGP@Se with significant antiproliferative activity against cancer cells *in vitro*. Future research should explore the mechanisms of action and validate these findings in animal models to advance TBGP@Se towards clinical applications in cancer treatment.

## Abstract 2

Tuna-bone gelatin peptides formed stable selenium complexes confirmed by FTIR, DLS, and SEM. TBGP@Se reduced viability of A549 and HT-29 cells in a dose-dependent manner, highlighting its anticancer potential.

## Graphical abstract

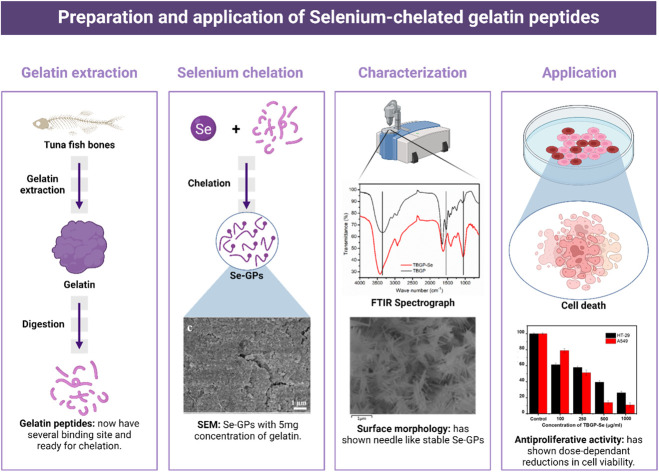

## 1. Introduction

Inorganic selenium (Se) is one of the most important trace bioelements and it is crucial for various metabolic pathways.^1^ Specifically, Se is used as a precursor for selenoproteins which have significant antioxidant effects.^2^ Inorganic Se has garnered significant attention as anti-cancerous and antioxidant agent^3^ owing to its potential in eliminating tumor growth *via* DNA repair and cell cycle regulation.^4^ For example, Se, in the form of selenite at 2.5 µM concentration, induces apoptosis *via* upregulation of p53 genes, thereby inducing cell cycle arrest and cell death in prostate tumor cells.^5^ Moreover, Se alters cancer cell metabolism *via* downregulating tumor growth proteins thus reducing cancer viability.^6^ Despite this significance, challenges, such as high off-target toxicity, non-targeted delivery, impenetrability across biological barriers and thus lower bioavailability remain to be investigated. Although, the trace amounts of Se have demonstrated promise in cancer treatment, the inherent instability of pure Se further complicates its application as an anticancer agent^7^ which further necessitates the development of more stable and less toxic formulations.

In recent years, research has shown another approach of bioconversion of Se to organic Se *via* selenization of carbohydrates or proteins which can achieve a more stable molecular structure while retaining its targeted therapeutic activity against tumor cells.^8–10^ These chelated forms not only mitigate the off-target toxicity of Se but also enhance its bioavailability and efficacy as an anticancer agent. For instance, organic forms of Se such as seleno-aspirin has shown promise in prostate cancer cell lines as its persistent exposure arrests the cell cycle in G1 phase further inducing caspase-dependent apoptotic pathway.^11^ This bioconversion warrants the importance of finding suitable carriers for Se to maximize its therapeutic potential.

On the other hand, gelatin, known for its antioxidant, anticancer, antibacterial, and antihypertensive properties,^12^ is valuable in the food and pharmaceutical industries due to its low cost, biocompatibility, non-toxicity, and biodegradability.^13^ Chemically, gelatin is a denatured protein from sources like bovine and fish, capable of cross-linking with other materials and contains a mix of anionic, cationic, and amphoteric groups.^14^ Henceforth, gelatin-based nanoparticles grafted with Se for cancer treatment with improved efficacy and stability are yet to be understood.

**Fig. 1:**
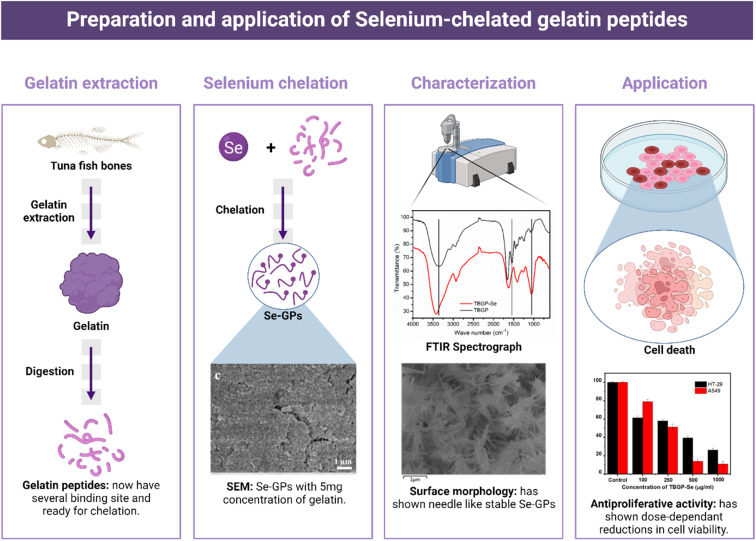
Schematic of how selenium was used to modify gelatin peptides for potential use in antiproliferative applications.

To address this gap, we, herein, aim to formulate stable and less toxic novel selenium-chelated tuna bone gelatin peptides (TBGP@Se) by simple redox reaction of sodium selenite and ascorbic acid with mild conditions. We demonstrate a green chemistry synthetic method of functionalized TBGP@Se which are hypothesized to offer a stable and effective delivery system with promising potential for tumor-targeted applications (Fig. 1).

## 2. Material and methods

### 2.1. Materials

**Fig. 2:**
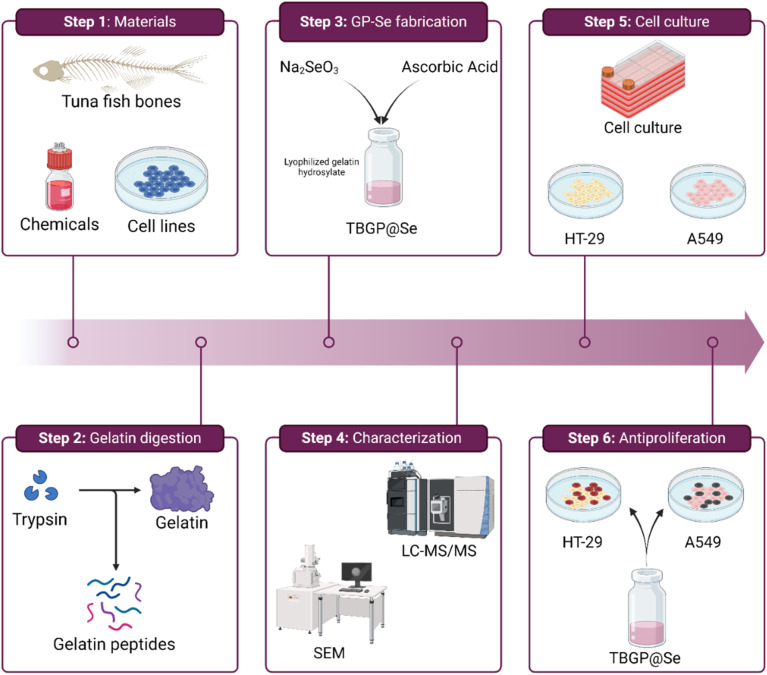
Series of experiments performed hereafter. The methods were divided into 6 steps from gelatin preparation to its characterization followed by gelatin peptide fabrication with selenium, and its antiproliferative activity on cell lines.

Tuna bones were supplied by Pak Aqua Industries (Karachi, Pakistan). Sodium selenite (Na_2_SeO_3_), Vitamin C, pepsin, trypsin from bovine pancreas, phosphate buffer saline (PBS, pH 7.4), ammonium hydrogen carbonate (NH_4_HCO_3_), Human pulmonary carcinoma A549 cell line, and human colon adenocarcinoma HT-29 cell line were purchased from Sigma-Aldrich. All other reagents used were of analytical grade. The whole experiment is illustrated in Fig. 2.

### 2.2. Preparation of tuna bone gelatin peptides (TBGP)

Gelatin was extracted by enzymatic method. Gelatin was dissolved in 50 mM ammonium hydrogen carbonate (1 : 10 w/v). The mixture was pretreated at 37 °C and trypsin was added at a 1 : 100 w/v of enzyme-gelatin ratio. The temperature of the mixture was maintained at 37 °C for 12–16 hours to facilitate the hydrolysis. Subsequently, the mixture was heated at 90 °C for 10 minutes to inactivate the enzyme. The hydrolysate was centrifuged at 10 000 rpm for 10 minutes, and the supernatant was collected and lyophilized for further use.

### 2.3. Fabrication of tuna bone gelatin peptides@selenium (TBGP@Se)

The lyophilized peptide solutions of different concentrations (0–10 mg mL^−1^) were mixed with freshly prepared 20 mM sodium selenite solution (1 : 1 v/v) under constant stirring, then 100 mM ascorbic acid (1 : 4 v/v) was added dropwise into the mixture. After that the mixture was put into the dark for 8 hours with constant stirring at room temperature. The solution was centrifuged at 10 000 rpm for 10 minutes, washed with deionized water, and freeze dried.

### 2.4. Characterization of TBGP & TBGP@Se

#### 2.4.1. Amino acid composition of gelatin

The amino acid composition of tuna bone gelatin (TBG) was determined by the method reported by D. Ding *et al.*, 2019.^15^ The TBG was treated with 6 M HCl and purged with nitrogen for 6 minutes. After evacuation of gas, the TBG was hydrolyzed for 24 hours at 110 °C. The hydrolysate was analyzed by Hitachi L-8900 high speed amino acid analyzer. The total content of each amino acids were expressed as number of residues/1000 residues.

#### 2.4.2. LC-MS/MS for peptide sequence

The peptide sequences were determined by LC-MS/MS. The TBGPs were separated by gradient elution at a flow rate of 0.250 µL min^−1^ with a Thermo-Dionex Ultimate 3000 HPLC system coupled with Thermo LTQ-Orbitrap Velos Pro mass spectrometer. Mobile phase A was consisted of 0.1% formic acid, while mobile phase B was containing 99.9% acetonitrile with 0.1% formic acid. The mass spectrometer was operated in data-dependent acquisition mode and scanned in the range of 400–2000 *m*/*z*, 30 000 resolutions.

#### 2.4.3. Analytical characterization

The surface morphology of TBGP@Se was observed by SEM (Jeol JSM-IT500). The solid sample was spread out and attached to the specimen holder by double sided adhesive tape and sputter-coated with a about 10 nm thick gold layer to increase conductivity. SEM images were taken at an accelerating voltage of 5.0 kV and working distance of 8.0 mm. The elemental analysis of TBGP@Se was analyzed by EDS spectrometer. The infrared spectra of TBGP, and TBGP@Se were characterized according to KBr pallet method by Fourier transform infrared spectrophotometer (FTIR) (PerkinElmer Spectrum) in the range of 400–4000 cm^−1^ with a spectral resolution of 4 cm^−1^. The UV-vis spectra were carried out on spectrophotometer (Shimadzu UV-2600) in a 1 cm quartz cuvette from 200 to 800 nm. While, DLS and zeta potential was measured on particle analyzer (Malvern Zetasizer). For DLS, hydrodynamic diameter of samples was measured at an angle of 173° at room temperature.

### 2.5. Cell culture

The human lung adenocarcinoma epithelial cell line A549 and the human colorectal adenocarcinoma cell line HT-29 were cultured in a RPMI-1640 medium including 10% of fetal bovine serum (FBS), 50 µg mL^−1^ of streptomycin, and 50 U ml^−1^ of penicillin at 37 °C under 5% CO_2_.

### 2.6. Antiproliferation activity

The cell culture and treatment were investigated using a previously described method.^16^ In detail, cell cytotoxicity was measured by using a tetrazolium salt WST-8 (Cell Counting Kit 8, CCK-8). A549 and HT-29 cells (6 × 10^3^ per well) were pretreated with the sample at different concentrations in a 96-well plate for 24 h. After that, 10 µl of WST-8 was added and the mixture was incubated at 37 °C for 2 h. Cell viability was calculated in the light of the absorbance at 450 nm by SpectraMax M2/M2e (CA, USA).

### 2.7. Statistical analysis

All experiments were performed in triplicate, and the results are presented as mean ± standard deviation (SD). Statistical significance was evaluated using one-way analysis of variance (ANOVA) followed by Tukey's post hoc test, with *p* < 0.05 considered significant. Data analysis and graph preparation were carried out using GraphPad Prism 9.0 (San Diego, CA, USA) and OriginPro 2021 (OriginLab, Northampton, MA, USA).

## 3. Results and discussion

### 3.1. Amino acid composition

**Fig. 3:**
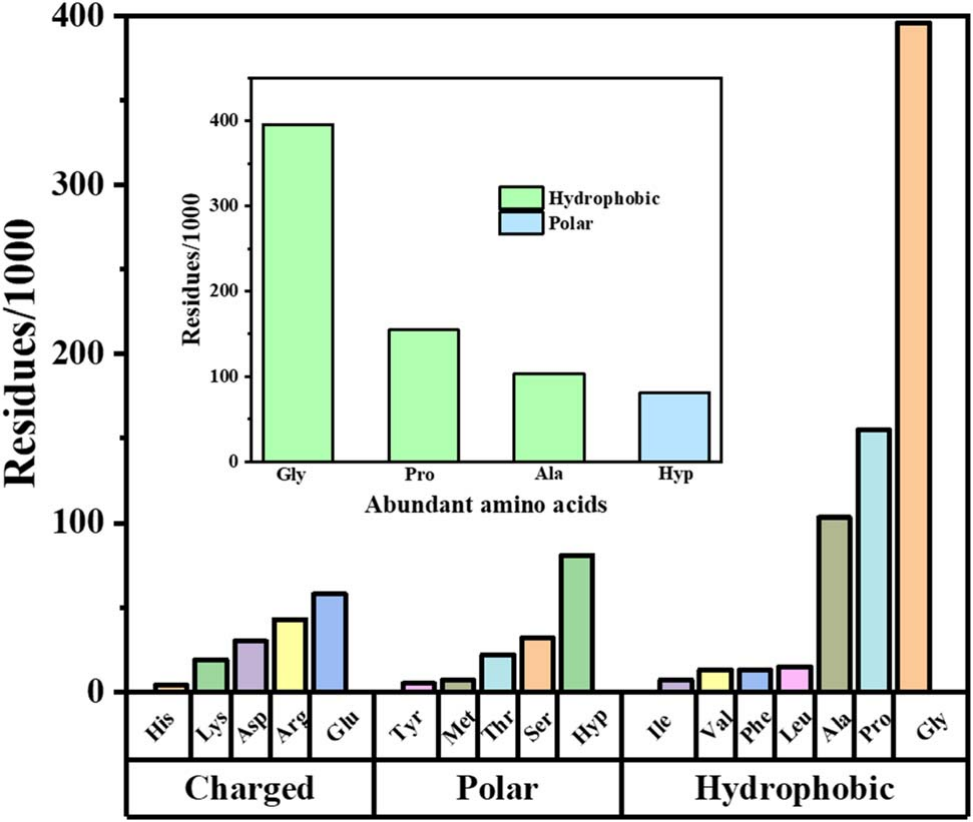
Amino acid composition of gelatin and their relative abundance. Out of total 17 amino acids, we categorized them into hydrophobic, charged and polar amino acids and out of them, charged and polar amino acids showed least abundance, while hydrophobic amino acids were comparatively higher. Glycine, proline, alanine and hydroxyproline were showed most abundance which potentially influences the structure of gelatin and facilitates chelation with Se (inside figure).

The amino acid composition analysis of tuna bone gelatin (TBG) (as depicted in Fig. 3) revealed that glycine (396 residues/1000) was the most abundant amino acid which contributes flexibility and ease of interaction with selenium (*p* < 0.01, compared to other amino acids). Similar proportion was observed in gelatin extracted from skipjack tuna scales^17^ and tuna bones.^18^ In our study, proline (Pro) and alanine (Ala) were also present in substantial amounts, with 155 and 103 residues per 1000 residues, respectively (*p* < 0.01 *vs.* other amino acids except glycine). Hydroxyproline (Hyp) was found at 81 residues per 1000 residues (*p* < 0.05, significant compared to Ala and Arg). Pro and Hyp are significant players in maintaining triple helical structure of gelatin, whereby, additional hydroxyl group in hydroxyproline assist reinforcing triple helical structure thus allowing extensive hydrogen bonding within gelatin structure.^19^ This offers a rigid and well-organized framework that could facilitate the integration of selenium atoms and makes the gelatin an effective carrier for selenium.

Literature survey revealed varying distribution of these imino acids (Hyp and Pro), for instance, 177.3/1000 residues in tuna bones^18^ and 183/1000 residues in *Solea vulgaris* skin 20, however, bovine gelatin showed higher proportion of these imino acids (219/1000 residues).^21^ Although the proportions of these imino acids vary across different gelatins, the amounts presented in our study suggests that the gelatin used is structurally competent for effective Se chelation.

Finally, other amino acids included glutamic acid (Glu) with 58 residues and arginine (Arg) with 43 residues per 1000 residues (*p* < 0.05, compared to low-abundance residues such as His, Tyr, Met, Ile). Interestingly, the carboxyl groups of Glu and the guanidinium group of Arg can form stable coordination complexes with selenium^22^ that supports the binding of Se within the gelatin matrix. Moreover, the presence of Glu and Arg contribute to the overall negative charge of the gelatin, which can attract and stabilize positively charged selenium species through ionic interactions. Similar roles of acidic residues in metal coordination have been highlighted in metalloproteins and selenium-binding proteins, where Glu residues contribute significantly to redox activity and metal–ligand stabilization.^23^

In contrast, histidine (His), tyrosine (Tyr), methionine (Met), and isoleucine (Ile) were found in relatively low amounts, ranging from 4 to 7 residues per 1000 residues (*p* < 0.01, significantly lower than Gly, Pro, and Ala). These low levels not only suggest that these residues do not significantly interfere with the chelation process but also the low content of these less polar or hydrophobic amino acids helps maintain the flexibility and solubility of gelatin, which is favorable for forming a homogeneous chelate with selenium. These findings provide a summary profile of amino acid composition of TBG.

### 3.2. Peptide sequencing

The gelatin obtained from tuna bones was hydrosylated into peptides which makes the gelatin binding sites more exposed for selenium binding. The identification of peptide sequences in TBGPs was achieved using liquid chromatography-tandem mass spectrometry (LC-MS/MS) with separation facilitated by the Thermo-Dionex Ultimate 3000 HPLC system coupled with the Thermo LTQ-Orbitrap Velos Pro mass spectrometer. The identified peptide sequences in TBGPs, offer insights into the complex composition and structural diversity of gelatin peptides. The analysis revealed several distinct peptide sequences, such as GETGPSGPAGPAGVAGAR (*m*/*z* 754.8781, RT 16.88), TVGLLSPR (*m*/*z* 421.759, RT 18.13), and GSPGASGIAGAXGFXGAR (*m*/*z* 779.8859, RT 19.09) (Table 1). In contrast to the retention time (16.88 to 32.69 minutes) of peptides in our study, Zhang *et al.*, 2019 (ref. 24) demonstrated a narrower range of retention times ranging from 9.05 to 17.22 minutes indicating a greater capacity for binding or interacting with selenium ions. Our larger peptides allow more functional groups available for selenium. Moreover, peptides in our study, for instance, P1 (GETGPSGPAGPAGVAGAR) and P4 (GETGPAGISGPAGPAGPR) contain multiple Pro and Gly residues, which contribute to a stable, flexible structure that can effectively chelate selenium. Proline, found abundantly in TBGP sequences such as GETGPSGPAGPAGVAGAR and GSPGASGIAGAXGFXGAR, introduces conformational rigidity while simultaneously conferring local flexibility through kink-inducing motifs.^25^ This structural effect facilitates the spatial orientation of functional groups, thereby exposing carboxyl and amide groups for selenium binding. Such proline-mediated conformational adaptations are consistent with previous findings that imino acid-rich peptides stabilize polyproline-II helices and enhance solvent accessibility for metal chelation.^26^

**Table 1:** Most abundant peptide sequences of tuna bone gelatin peptides (TBGPs) identified by LC-MS/MS, including mass-to-charge ratio (*m*/*z*), retention time (RT), and mass

Sr	Peptide sequence	*m*/*z*	*z*	RT (min)	Mass
P1	GETGPSGPAGPAGVAGAR	754.8781	2	16.88	1507.738
P2	TVGLLSPR	421.759	2	18.13	841.5021
P3	GASGTXGVAGAXGFXGPR	800.8914	2	18.34	1599.764
P4	GETGPAGISGPAGPAGPR	774.8939	2	18.46	1547.769
P5	GSPGASGIAGAXGFXGAR	779.8859	2	19.09	1557.754
P6	DGM(+15.99)NGLXGPIGPXGPR	790.3825	2	22.52	1578.746
P7	GDVGPXGLTGFXGAAGR	779.3885	2	24.21	1557
P8	GLTGPIGLXGXAGATGDK	805.925	2	25.46	1609.831
P9	VQAGPAGLVGPXGPXK	737.4094	2	32.69	1472.799
P10	NGPLSDMLVM(+15.99)MM(+15.99)VGFLEGGK	720.0206	3	26.91	2156.998
P11	EGXGPAGLLGPAQR	668.3558	2	20.76	1334.694
P12	EGTGAFGPAGPAGPR	671.3327	2	18.51	1340.647

Additionally, the presence of Arg in several sequences can enhance electrostatic interactions with selenium. This is in contrast to Zhang *et al.*, 2019, such as WMFDW and WMGPY, contain aromatic and hydrophobic residues that are less likely to contribute to chelation.^24^ Furthermore, Arg, with its guanidinium group, forms strong electrostatic and hydrogen-bonding interactions with selenium species. The role of basic residues such as Arg in stabilizing negatively charged oxyanions, including selenite and selenate, has been established in structural studies of selenoproteins and transporters.^27^

Furthermore, the smaller size of peptides (608.60 Da) as demonstrated by Qiu *et al.*, 2019 (ref. 17) from *Katsuwonus pelamis*, may limit their ability to form stable structures compared to our larger peptides such as P1 and P4 with masses around 1500–1600 Da. Peptides from Qiu *et al.*, 2019 (ref. 17) contain Glu and His, both of which are important for metal binding. However, the lower number of these residues per sequence could result in fewer binding sites for selenium. In contrast to it, Glu in our sequences, such as P6 (DGM (+15.99) NGLXGPIGPXGPR) provides carboxyl groups that can participate in selenium ion chelation. The mass-to-charge ratios (*m*/*z*) and retention times (RT) for our peptides were consistent with their calculated masses which thus confirms their identities and provides insights into the composition and structure of the gelatin peptides.

**Fig. 4:**
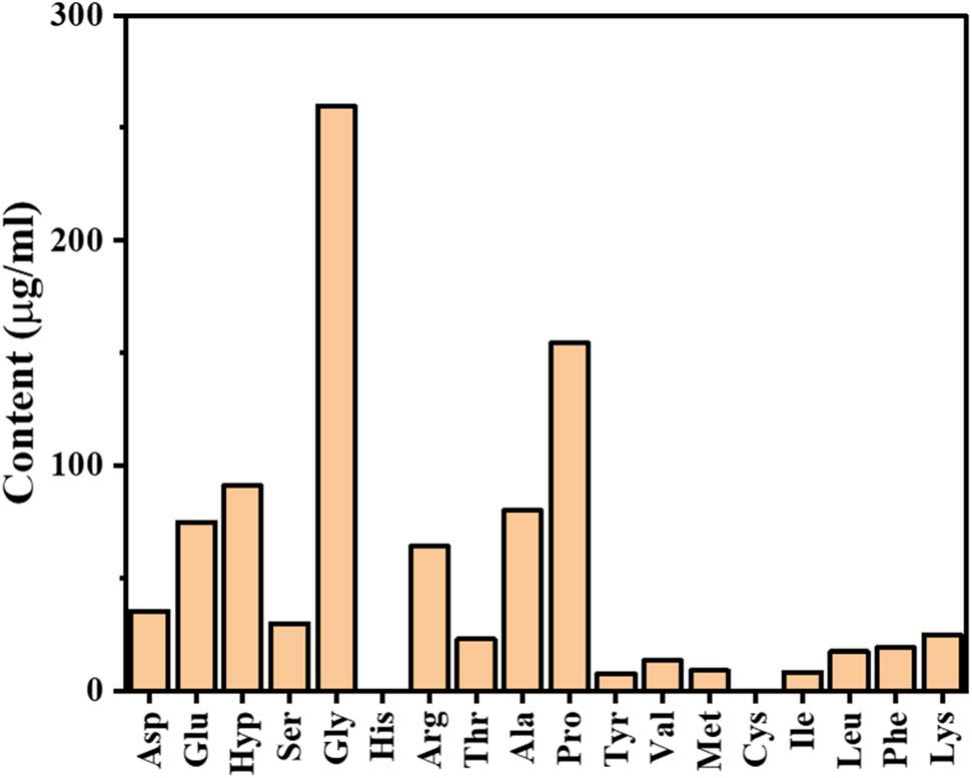
Chromatography peak analysis of amino acids. The concentration of each amino acid was measured in µg mL^−1^. Notable concentrations include Gly at 259.9 µg mL^−1^, Pro at 154.4 µg mL^−1^, and Glu at 75 µg mL^−1^. Some amino acids, like His and Cys, have a content of 0 µg mL^−1^ as these amino acids were undetectable.

Further chromatographic peak analysis was used to measure the retention times and concentrations of various amino acids in the gelatin samples (Fig. 4). Again, glycine was the most abundant amino acid, with a retention time of approximately 9.2 minutes and a concentration around 0.26 mg mL^−1^ in both gelatin samples. Other significant amino acids included proline (RT 13.7 minutes, ∼0.16 mg mL^−1^), alanine (RT 12.8 minutes, ∼0.08 mg mL^−1^), and hydroxyproline (RT 6.7 minutes, ∼0.09 mg mL^−1^) highlight their prominence in the gelatin structure. This detailed characterization of peptide sequences and amino acid composition provides a comprehensive understanding of the structural and functional properties of TBGPs. These sequences, such as GETGPSGPAGPAGVAGAR and GSPGASGIAGAXGFXGAR, highlight specific patterns and arrangements within the gelatin structure. The proline acts as a molecular hinge that induces kink-swivel motions in peptides and decouples helix portions.^28^ Interestingly, proline-rich peptides with glycine/alanine insertions exhibit stabilized polyproline-II conformations which not only influences the conformational transitions but also the stability of the peptides.^29^ Therefore, in our study, in TBGPs, the presence of proline, glycine, and alanine in the identified sequences suggests that similar structural dynamics and stabilization mechanisms may be at play.

### 3.3. Characterization of TBGP and TBGP@Se

**Fig. 5:**
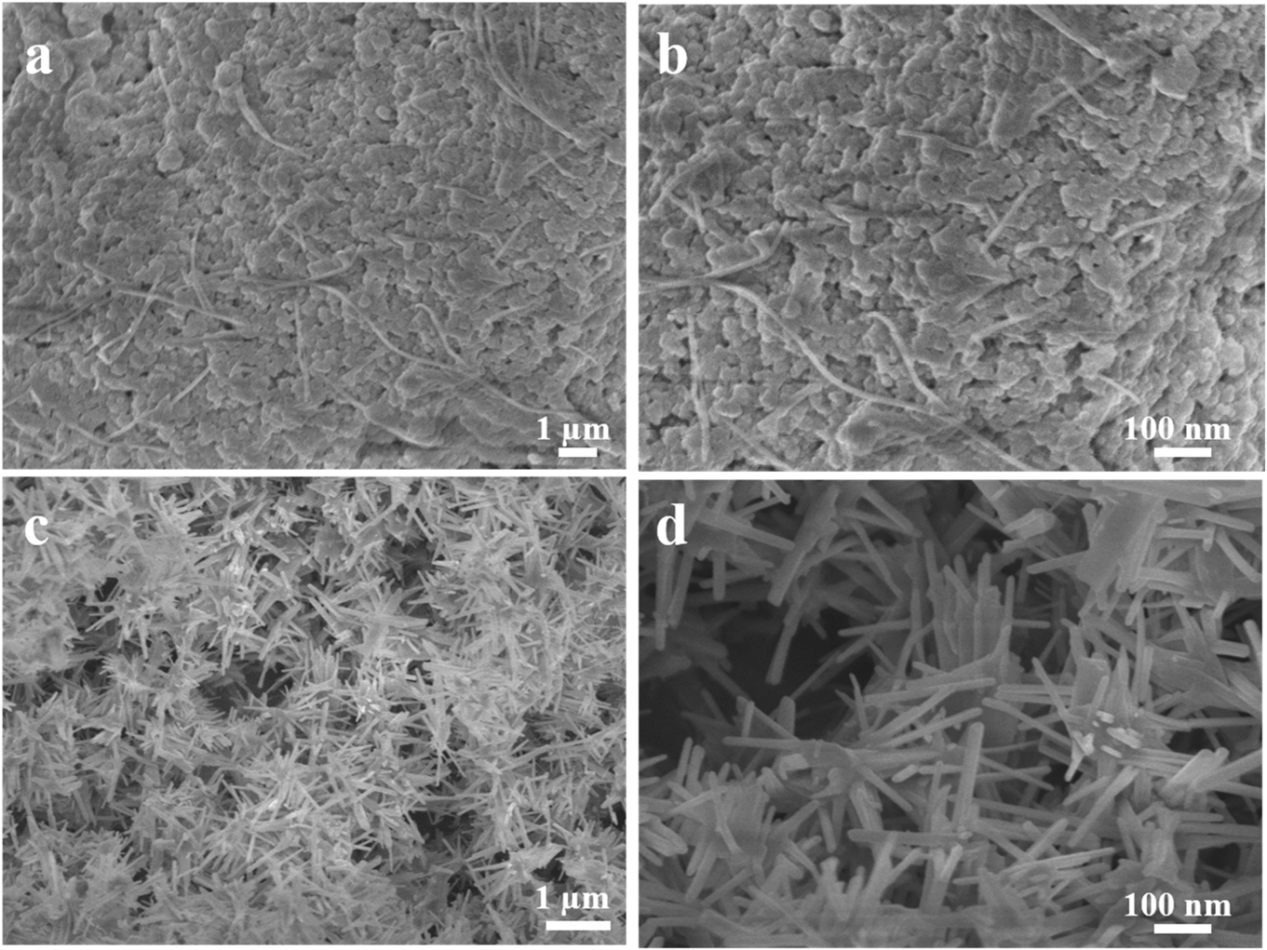
Scanning electron microscopy (SEM) analysis of TBGP@Se complexes at 5 mg mL^−1^ (a and b), uniform and compact nanostructures are formed with minimal aggregation, indicating an optimal peptide-to-selenium ratio that produces stable complexes and selenium nanorods (SeNR) (c and d).

To evaluate the stability of TBGP@Se, various concentrations of digested gelatin (1 mg mL^−1^, 3 mg mL^−1^, 5 mg mL^−1^, 7 mg mL^−1^, and 10 mg mL^−1^) with selenium were analyzed using SEM (Fig. 5 and S1). As shown in (Fig. 5a and b) 5 mg mL^−1^ concentration of gelatin produced the most stable TBGP@Se complexes. This indicates that at intermediate gelatin concentrations, more binding sites were available for selenium chelation, thereby leading to greater stability and more efficient incorporation of Se into the gelatin matrix. Thus, in next procedures, we used this concentration.

The surface morphology of the tuna bone gelatin peptides–selenium (TBGP@Se) was observed using scanning electron microscope. The samples were prepared by sputter-coating with gold to enhance conductivity and improve image resolution. SEM images revealed a distinctive wires-like structural appearance, indicating a well-defined and uniform morphology of the Se-TBGP complex. While selenium particles represented needle like morphology without addition of gelatin peptides.

**Fig. 6:**
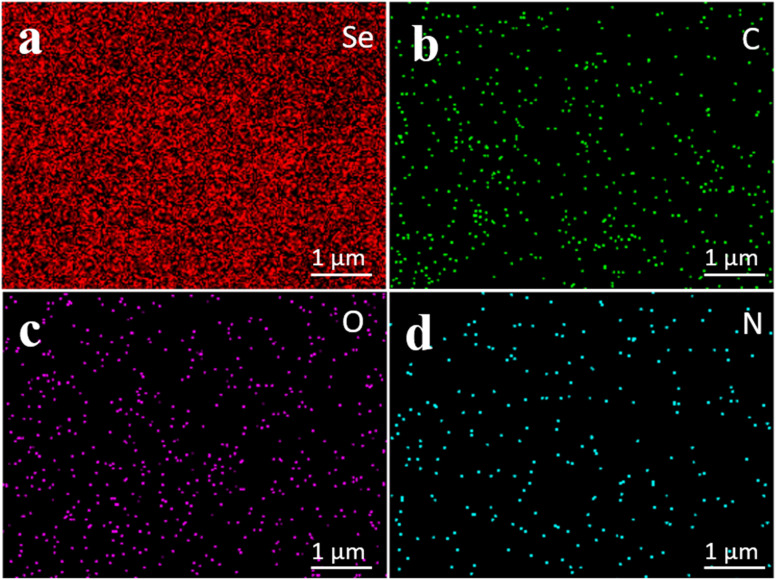
Energy dispersive X-ray spectroscopy images of TBGP@Se at 5 mg mL^−1^ concentration. Three parameters such as, uniformity, concentration and elemental composition, were interpreted from these images. (a) Selenium was 40% in the TBGP matrix with uniform distribution and higher concentrations at various areas. (b) Carbon, (c) oxygen and (d) nitrogen showed least concentrations however, uniform patterns of dispersion.

Additionally, elemental composition analysis using Energy Dispersive X-ray Spectroscopy (EDS) provided quantitative data on the atomic percentages of elements present in the TBGP@Se samples as shown in Fig. 6. The results showed that carbon (C) constituted 51.10% of the total atomic content (Fig. 6b), oxygen (O) was 8.38% (Fig. 6c), and selenium (Se) made up a significant portion at 40.52%. Nitrogen (N) was in trace amounts in the samples (Fig. 6d). Moreover, Se was detected to be uniformly distributed throughout the TGBP matrix which further confirms even integration of selenium within TGBP structure Fig. 6a. This uniform distribution of selenium shows promises that TBGP can serve as an effective delivery vehicle for selenium. Nonetheless, the concentration of higher intensity areas indicates higher local intensity of Se. These findings confirm the successful incorporation of selenium into the gelatin peptides thus contributing to the unique wire-like morphology observed in the SEM images.

**Fig. 7:**
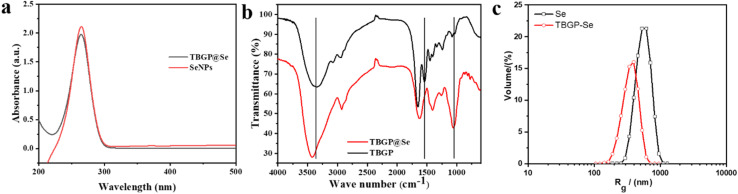
Characterization of TBGP@Se complexes compared to TBGP alone. (a) UV-vis absorption spectra showing a distinct absorption peak shift upon selenium incorporation into TBGP, confirming successful chelation and interaction between selenium and peptide residues. The TBGP@Se complexes (red line) display a strong peak in the 250–300 nm range, characteristic of Se-related electronic transitions, whereas TBGP alone (black line) shows no such prominent peak. (b) FTIR spectra demonstrating structural and functional group changes associated with selenium binding. The TBGP@Se complexes (red line) show shifts in amide I (C=O stretching) and amide II (N–H bending) bands relative to TBGP alone (black line), indicating coordination of selenium with carboxyl and amine groups from peptide side chains (notably Glu and Arg residues). Additional spectral modifications suggest conformational stabilization of gelatin after selenium chelation. (c) Dynamic light scattering (DLS) analysis revealing the hydrodynamic size distribution of TBGP@Se complexes (red dots) compared to TBGP (black dots). TBGP@Se exhibits a sharp, monodisperse peak centered around ∼200 nm, significantly larger than TBGP alone (∼50–70 nm), confirming nanoparticle formation and successful self-assembly of selenium within the peptide framework.

The optical properties of TBGP@Se were assessed using UV-visible spectroscopy. An absorbance peak was observed at 265 nm, confirming the successful formation of nanoparticles (Fig. 7a). This result is consistent with prior research on selenium–lysozyme nanohybrid systems, which also demonstrated a characteristic absorption peak at 265 nm.^30^ In our case, the absorption intensity of TBGP@Se was slightly reduced when compared to selenium nanoparticles (Se-NPs), a trend that aligns with similar findings reported by Vahdati and Moghadam (2020).^30^ This subtle decrease in absorbance could be indicative of changes in nanoparticle morphology or interaction between TBGP and selenium, which may affect the overall optical behavior. These observations suggest that the conjugation of TBGP with selenium could modulate the absorbance properties of the nanoparticles, contributing to their unique characteristics.

The FTIR and DLS analyses provided clear evidence of successful incorporation of selenium into tuna bone gelatin peptides (TBGP). FTIR spectra revealed distinct spectral features for TBGP, and TBGP chelated with selenium (TBGP@Se) (Fig. 7b). The shifts in the characteristic absorption peaks in the FTIR spectra provide valuable insights into the molecular interactions between selenium and the organic ligand groups of TBGP. As shown in Fig. 7b, the absorption peak of TBGP at 3400 cm^−1^, corresponding to the –NH_2_ group in the amide-A band, exhibited a blue shift to 3430 cm^−1^ after conjugation with selenium in the TBGP@Se complex. This shift suggests alterations in the chemical environment of the –NH_2_ group. Additionally, the FTIR spectra of TBGP revealed a strong absorption band at 1657 cm^−1^, which is attributed to the stretching vibrations of C=O bonds (amide I). After chelation with selenium, this band transformed into two weaker absorption peaks, indicating structural changes in the TBGP molecule upon interaction with selenium. These changes imply that selenium binding significantly affects the stretching vibrations of the –NH group, highlighting the involvement of both –NH_2_ and –NH groups as key sites for selenium chelation. Moreover, the evidence points to the carboxylate group playing an essential role in the coordinate binding during the chelation process. The interaction between selenium and TBGP likely involves complex coordination with both amide and carboxylate functional groups. Thus, the chelation between TBGP and selenium is primarily driven by the reactive sites of amides and carboxylates, which are crucial in forming a stable complex. Whereas, Ye *et al.* (2019) noted shifts in the amide-A band and splitting of the amide I band in their selenium–polysaccharide interactions.^31^ Additionally, Ye *et al.* (2019) found that chelating selenium with soybean peptides resulted in a folded and aggregated structure which enhanced its antioxidant activity and reducing oxidative damage in cells.^31^ DLS analysis further demonstrated a reduction in particle size distribution for TBGP@Se (PDI = 0.495) compared to selenium particles (PDI = 0.727) (Fig. 7c) which suggests an enhanced dispersion and stability of selenium when chelated with gelatin peptides. Furthermore, both have negative zeta potential values but TBGP@Se has more negative potential as compared to selenium articles due to presence of abundant carboxylate group on the surface. These findings confirm that selenium was successfully integrated into the gelatin matrix of selected concentration and formed stable and uniformly sized TBGP@Se complexes.

### 3.4. Antiproliferation activity

**Fig. 8:**
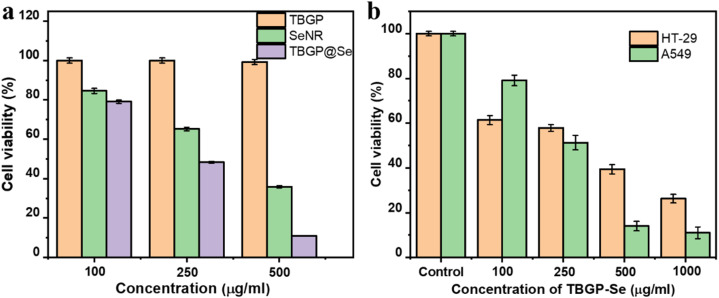
Antiproliferation activity of TBGP, selenium nanorods, and TBGP-Se. (a) TBGP alone did not show any antiproliferative effect (100% viability) at all tested concentrations (100 µg mL^−1^, 250 µg mL^−1^, and 500 µg mL^−1^) (*n* = 3, *p* > 0.05 *vs.* control). In contrast, selenium nanorods (SeNR) displayed decreasing viability with increasing concentration: 85% at 100 µg mL^−1^, 65% at 250 µg mL^−1^, and 30% at 500 µg mL^−1^ (*n* = 3, *p* < 0.01 *vs.* control). TBGP-Se exhibited more potent effects: 78% viability at 100 µg mL^−1^, 50% at 250 µg mL^−1^, and 10% at 500 µg mL^−1^ (*n* = 3, *p* < 0.001 *vs.* TBGP and SeNR). (b) Comparative analysis of TBGP-Se against HT-29 and A549 cell lines was employed. Cell viability percentages are shown after treatment with TBGP-Se at concentrations of 100, 250, 500, and 1000 µg mL^−1^. Control groups for both cell lines exhibited 100% viability (*n* = 3). TBGP-Se treatment resulted in significant dose-dependent reductions in viability: at 1000 µg mL^−1^, viability decreased to 25% in HT-29 and less than 10% in A549 cells (*n* = 3, *p* < 0.001 *vs.* control).

Upon analyzing the data from cell viability assays in A549 cell lines treated with TBGP, selenium NR, and TBGP@Se complexes, distinct trends emerged. TBGP exhibited consistently high cell viability across all tested concentrations (100, 250, and 500 µg mL^−1^) which demonstrates its minimal cytotoxicity on A549 cells (Fig. 8a) (*n* = 3, *p* > 0.05 *vs.* control). In contrast, selenium NR displayed a dose-dependent decrease in cell viability which indicate increased cytotoxicity with higher concentrations (*n* = 3, *p* < 0.01 compared to TBGP and control). The TBGP@Se resulted in strong effects on cell viability compared to TBGP alone and selenium NR (*n* = 3, *p* < 0.001). Specifically, TBGP@Se complexes showed higher anti-cancerous activity than TBGP and selenium nanorods, particularly at higher concentrations.

As indicated above, 5 mg mL^−1^ TBGP@Se showed more stability, we ran a comparative cell viability analysis with 1 mg mL^−1^, 3 mg mL^−1^, 7 mg mL^−1^, and 10 mg mL^−1^ (Fig. S2) to check the antiproliferative activity (*n* = 3, *p* > 0.05 compared to 5 mg mL^−1^). The findings were coherent owing to least effects with these concentrations as compared to 5 mg per mL TBGP@Se.

The antiproliferative activity of TBGP@Se was then evaluated on A549 in comparison with HT-29 cell lines. The viability of both cell lines decreased progressively with increasing concentrations of TBGP@Se. In comparison with control group, the increased concentration of TBGP@Se caused a significant reduction in cell viability in both cell lines (*n* = 3, *p* < 0.001 *vs.* control). This indicates that TBGP@Se has a dose-dependent effect on inhibiting cell proliferation. HT-29 cells generally exhibited higher sensitivity to TBGP@Se compared to A549 cells across all concentrations tested (Fig. 8b). At higher concentrations of TBGP@Se (500 µg mL^−1^ and 1000 µg mL^−1^), the decrease in cell viability was particularly pronounced which suggests a potent antiproliferative effect on both cell lines, albeit more prominently on HT-29 cells. This is similar to Li *et al.* (2021) whereby they used anticancer bioactive peptide-functionalized selenium (ACBP-S-Se) particles which effectively inhibited proliferation in gastric cancer cell lines (MKN-45 and MKN-74).^32^ Further, another study by Debnath and colleagues in 2022 (ref. 33) highlights potent cytotoxic effects of selenium-containing compounds against various cancer cell lines and underscores the potential of selenium-based treatments in cancer therapy. Similar to our TBGP@Se complexes, the combination of bovine lactoferrin and Se NPs demonstrated potent anti-proliferative effects on various cancer cell lines, including MCF-7, HepG-2, and Caco-2 with high selectivity against cancer cells while sparing normal cells.^34^

**Fig. 9:**
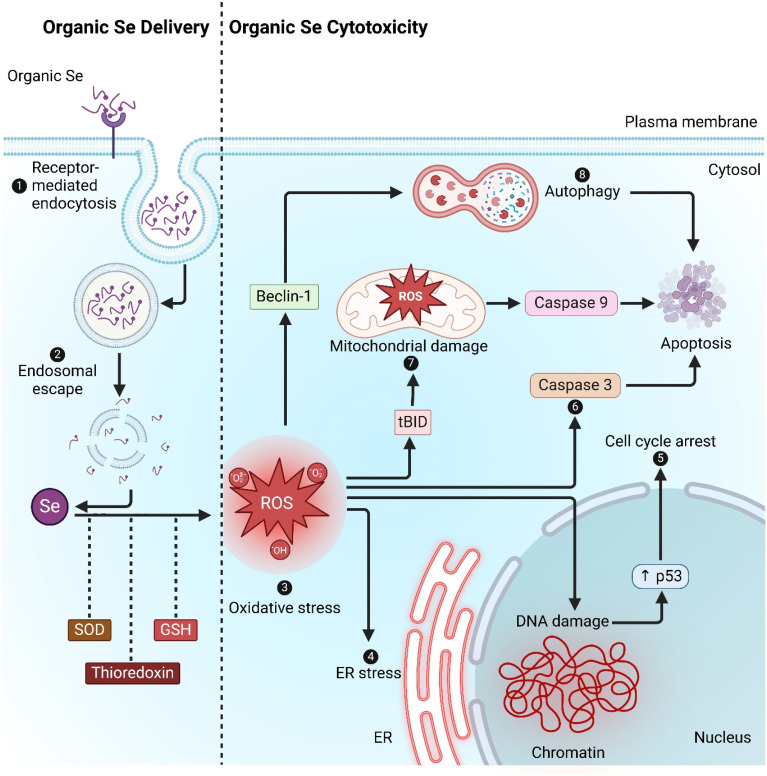
Cytotoxic mechanism of TBGP@Se in cancer cells. Based on existing literature on selenium compounds, the process involves receptor-mediated uptake of organic selenium, followed by endosomal escape into the cytoplasm. Selenium interacts with intracellular antioxidant systems, including superoxide dismutase (SOD), thioredoxin, and glutathione (GSH), leading to oxidative stress and the accumulation of reactive oxygen species (ROS). This triggers various cytotoxic effects: (1) ER stress activation, initiating apoptosis *via* intrinsic pathways, (2) DNA damage leading to p53 upregulation, cell cycle arrest, and apoptosis, (3) mitochondrial damage activating tBID and caspase-9, driving apoptotic cell death, (4) oxidative stress-induced caspase-3 activation, further promoting apoptosis, and (5) ROS-mediated upregulation of Beclin-1, contributing to autophagy-driven cell death.

The enhanced cytotoxicity observed with TBGP@Se complexes compared to TBGP or selenium nanorods alone can be explained through the well-established mechanisms of organic selenium-mediated anticancer activity. After entering the cells, organic selenium disrupts redox homeostasis. Selenium compounds interact with intracellular antioxidant defense systems such as superoxide dismutase (SOD), thioredoxin, and glutathione (GSH), which contribute to redox imbalance and the accumulation of ROS.^35^ Elevated ROS levels induce oxidative stress, thereby triggering multiple cytotoxic signaling cascades (Fig. 9).

ROS accumulation induces endoplasmic reticulum (ER) stress, activating apoptotic pathways through unfolded protein response mechanisms.^36^ Moreover, oxidative DNA damage promotes the stabilization and upregulation of p53.^37^ This results in cell cycle arrest and apoptosis. Mitochondrial dysfunction is also induced *via* ROS-mediated activation of pro-apoptotic proteins such as tBID, leading to cytochrome c release and caspase-9 activation, ultimately driving intrinsic apoptosis. Additionally, caspase-3 activation downstream of ROS further executes programmed cell death. Importantly, ROS can also upregulate Beclin-1, thereby promoting autophagy, which contributes to cancer cell death under conditions of sustained oxidative stress.^38,39^

Collectively, these interconnected mechanisms contribute to the potent antiproliferative and cytotoxic activity of TBGP@Se observed in A549 and HT-29 cells. Although our current study primarily employed cell viability assays, current literature strongly supports that selenium nanocomposites exert anticancer activity through ROS-mediated apoptosis and autophagy. Future studies with apoptosis assays (Annexin V/PI, caspase activity) and ROS quantification are warranted to validate these pathways in TBGP@Se systems.

Our study demonstrates promising potential in synthesizing TBGP@Se for *in vitro* antiproliferative activity, however, several limitations merit consideration. Firstly, while TBGP@Se showed significant cytotoxic effects, the underlying mechanisms are needed to be explored. Secondly, our findings represent initial *in vitro* evidence only in cancer cell lines, and further exploration of *in vivo* models in necessary. Lastly, our cell viability assays (WST-8) provide primary insights into cytotoxicity, further investigation into apoptosis, necrosis, or cellular signaling pathways involved in antiproliferative effects are suggested to be explore in future studies.

## 4. Conclusion

Our study on TBG peptides and its selenium-chelated complexes, TBGP@Se highlights significant findings across several fronts. Firstly, TBG is rich in glycine, proline, and alanine, which are crucial for its structural integrity. Peptide sequencing *via* LC-MS/MS identified specific sequences which reveal the diverse structural composition of TBGPs. SEM analysis revealed that higher concentrations of gelatin (5 mg mL^−1^) produced the most stable TBGP@Se complexes, crucial for selenium chelation. Importantly, TBGP@Se exhibited potent antiproliferative effects in A549 and HT-29 cell lines thereby underscoring its anti-cancer potential. These findings collectively advocate for further exploration of TBGP@Se as a promising bioactive compound in biomedical applications.

## Conflicts of interest

Authors declare no conflict of interest.

## Supplementary Material

RA-015-D5RA06829K-s001

## Data Availability

The authors confirm that all data supporting the findings of this study are available within the article. Supplementary information (SI) is available. See DOI: https://doi.org/10.1039/d5ra06829k.
