# Supplementary material for: Synthesis, characterization, and anticancer activity of tuna bone gelatin peptide-stabilized selenium nanowires
Source: RSC Adv. 2025 Dec 9;15(57):48958–68. doi: 10.1039/d5ra06829k (PMC12687417; doi:10.1039/d5ra06829k)
Supplement: RA-015-D5RA06829K-s001 [file RA-015-D5RA06829K-s001.pdf]

Supplementary Information (SI) for RSC Advances.
This journal is © The Royal Society of Chemistry 2025

Supporting information

for

Synthesis, Characterization, and Anticancer activity of Tuna Bone Gelatin Peptide-Stabilized Selenium Nanowires

Muhammad Babar Khawar[#], Kamaran Khursheed Dar[#], Ali Afzal[#], Aisha Munawar, Muhammad Waqas Ishaq, Fakhar Zaman[*]

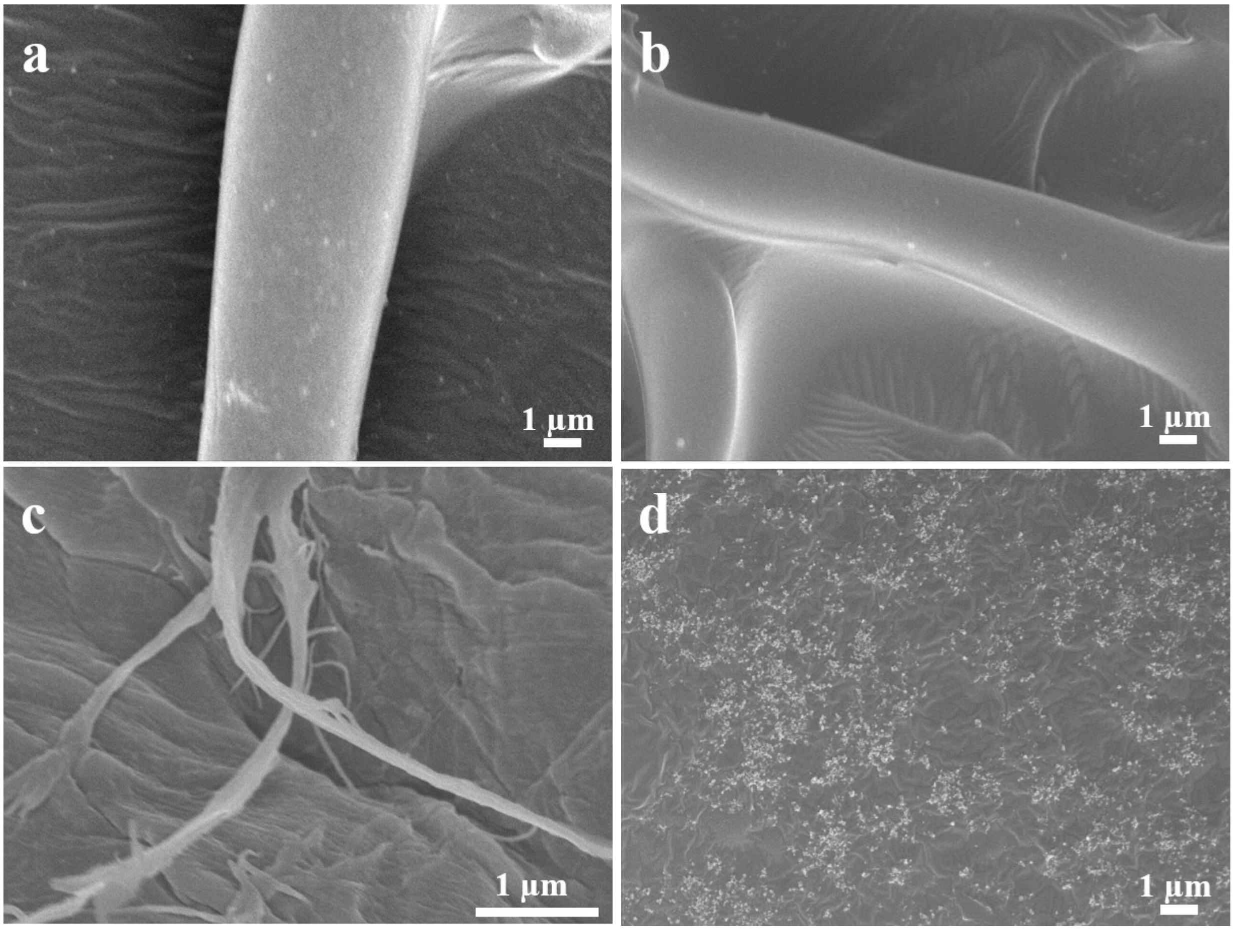

Figure S1: Scanning electron microscopy (SEM) analysis of TBGP@Se complexes at different concentrations. At 1 mg mL^{-1} (a) and 3 mg mL^{-1} (b), elongated fibrillary structures with partial aggregation are observed, suggesting incomplete stabilization of selenium species. At higher concentrations, 7 mg mL^{-1} (c) and 10 mg mL^{-1} (d).

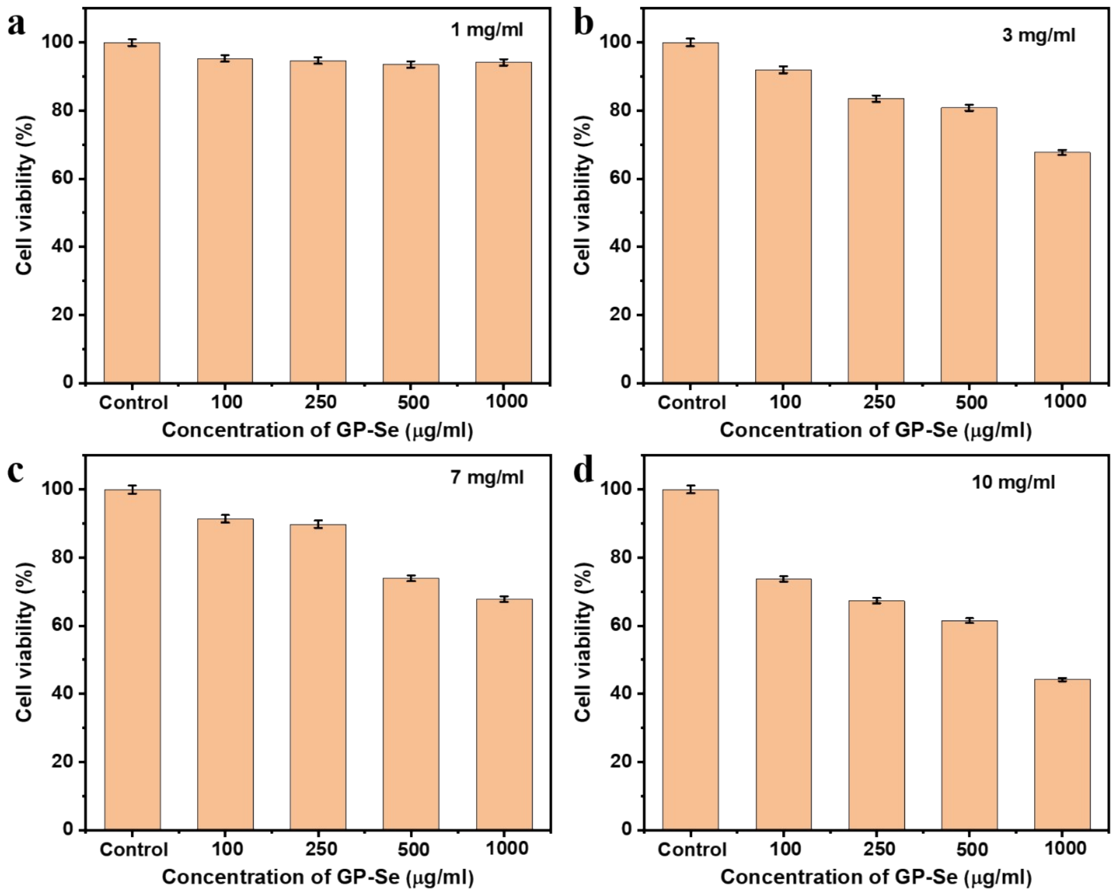

Figure S2: Antiproliferation activity of TBGP@Se against A549 at (a) 1, (b) 3, (c) 7, and (d) 10 mg/mL concentration.
